# A systematic identification of anti-inflammatory active components derived from Mu Dan Pi and their applications in inflammatory bowel disease

**DOI:** 10.1038/s41598-020-74201-x

**Published:** 2020-10-14

**Authors:** Tzu-Fan Chen, Jeh-Ting Hsu, Kun-Chang Wu, Che-Fang Hsiao, Jou-An Lin, Yun-Hsin Cheng, Yu-Huei Liu, Der-Yen Lee, Hen-Hong Chang, Der-Yang Cho, Jye-Lin Hsu

**Affiliations:** 1 Graduate Institute of Biomedical Sciences, China Medical University, Taichung, Taiwan; 2 Department of Information Management, Hsing Wu University, Taipei, Taiwan; 3 School of Pharmacy, College of Pharmacy, China Medical University, Taichung, Taiwan; 4 Graduate Institute of Integrated Medicine, China Medical University, Taichung, Taiwan; 5 Department of Medical Genetics and Medical Research, China Medical University Hospital, Taichung, Taiwan; 6 Drug Development Center, China Medical University, Taichung, Taiwan; 7 Translational Cell Therapy Center, Department of Medical Research, China Medical University Hospital, Taichung, Taiwan; 8 Department of Neurosurgery, China Medical University Hospital, Taichung, Taiwan

**Keywords:** Inflammation, Innate immune cells, Natural products, Gastrointestinal diseases

## Abstract

Mu Dan Pi (MDP), also known as Moutan Cortex Radicis, is a traditional Chinese medicine used to treat autoimmune diseases. However, the impact of MDP and its principal active compounds on inflammatory bowel disease (IBD) is uncertain. This study therefore systemically assessed the anti-inflammatory effects of MDP and its known active compounds in IBD. The anti-inflammatory activities of water extract and individual compounds were screened by NF-κB and interferon regulatory factor (IRF) reporter assays in THP-1 cells induced with either Toll-like receptor or retinoic acid inducible gene I/melanoma differentiation-associated gene 5 activators and further verified in bone marrow-derived macrophages. MDP water extract significantly inhibited the activation of NF-κB and IRF reporters, downstream signaling pathways and the production of IL-6 and TNF-α, in a dose-dependent manner. Among 5 known active components identified from MDP (1,2,3,4,6-penta-*O-*galloyl-β-*d*-glucose [PGG], gallic acid, methyl gallate, paeoniflorin, and paeonol), PGG was the most efficient at inhibiting both reporters (with an IC_50_ of 5–10 µM) and downregulating IL-6 and TNF-α. Both MDP powder for clinical use and MDP water extract, but not PGG, reduced colitis and pathological changes in mice. MDP and its water extract show promise as a novel therapy for IBD patients.

## Introduction

Inflammatory bowel diseases (IBD) are inflammatory disorders of the gastrointestinal tract that affect millions of individuals worldwide, at increasingly higher rates^1^. The two major disorders of IBD, Crohn’s disease (CD) and ulcerative colitis (UC), are characterized by both acute and chronic inflammation of the intestine with multifactorial etiology. Although the etiology of IBD remains largely unknown, many studies have shown that its pathogenesis involves a complex interaction between genetic, environmental/microbial factors, and immune responses^1^. Drugs for IBD, including aminosalicylates, corticosteroids and anti-tumor necrosis factor (TNF) therapies target different aspects of the immune response, but all are hampered by primary and secondary loss of response and have a number of contraindications and adverse effects^2–4^. It is therefore imperative that novel IBD drugs are developed using different strategies. Because innate immune cells such as macrophages and dendritic cells initiate the immune response against microbes and IBD is characterized by an aberrant innate-immune response^5,6^, this study focused on developing drug to regulate the most crucial signaling pathways triggered by microbes in innate immune cells.

Traditional Chinese medicine (TCM) has long been used in Asian countries to treat various inflammatory and autoimmune diseases^7^. In Taiwan, 70% of patients with psoriasis^8^, 27% of patients with rheumatoid arthritis^9^, and 37% of patients with IBD^10^ were TCM users. Of all TCMs, Mu Dan Pi (MDP) is one of the most commonly prescribed single herbs for the treatment of psoriasis and other inflammatory symptoms^8^. MDP, also known as Moutan Cortex Radicis, is a TCM derived from the root bark of *Paeonia suffruticosa* Andrews (Genus: *Paeonia*; Fam: *Paeoniaceae*)^11^. Its bioactive components exhibit anticancer, antimicrobial and antioxidant properties^12–16^ and are widely used for the treatment of diabetes, cancer, cardiovascular and neurological disorders^13,16,17^. The many components isolated from MDP with proven anti-inflammatory or immunomodulatory effects include paeonol, paeoniflorin, methyl gallate, gallic acid, and 1,2,3,4,6-penta-*O-*galloyl-β-*d*-glucose (PGG). Paeonol, the best-known active ingredient in MDP, has demonstrated cardioprotective effects in animal models, attenuated inflammatory and coagulation reactions after lipopolysaccharide (LPS)-induced acute lung injury^18^, and attenuated trinitrobenzene sulfonic acid (TNBS)-induced colitis. Paeoniflorin is the most abundant component and the main biologically active ingredient of the total glucosides of peony that is isolated from the roots of *P. lactiflora Pall*^17^. In animal models of human autoimmune diseases, including rheumatoid arthritis and systemic lupus erythematosus, paeoniflorin has demonstrated anti-inflammatory and immunoregulatory effects^17^. PGG is a polyphenolic bioactive constituent of medicinal plants such as *Paeonia suffruticosa, Rhus chinensis* Mill., *P. lactiflora* and mango (*Mangifera indica* Linn)^19^. Anti-inflammatory properties of PGG include its ability to ameliorate renal tubular injury and microvascular inflammation in a rat model of acute kidney injury^20^. However, it remains unclear as to which component most effectively contributes to the anti-inflammatory actions of MDP. Furthermore, whether MDP or its active components can be used for treating IBD remains to be discovered.

Human intestinal inflammation involves an innate response to pathogen-associated molecular patterns, such as peptidoglycan and lipoteichoic acid residing in bacteria cell walls. These molecular patterns can activate a recognition receptor, Toll-like receptor 2 (TLR2) on host cells and induce the activation of signal transduction cascades as well as double-stranded RNA derived from the viral genome, which can stimulate retinoic acid inducible gene I (RIG-I) or melanoma differentiation-associated gene 5 (MDA5). These ligands activate the NF-κB pathway, and/or activate interferon regulatory factors 3/7 (IRF 3/7), which cooperate to stimulate the transcription of various cytokines such as interferon alpha/beta (IFN-α/β), IL-6 and TNF-α, to counteract infection^21^. In addition to their anti-infective roles, high levels of IL-6 and TNF-α are found in inflammatory conditions and in IBD in particular^22^. This study screened various anti-inflammatory compounds from MDP in an attempt to identify which molecule most effectively mediates IRF and/or NF-κB signaling pathways. Applying different ligands with corresponding reporters could further offer the dissection of the TLR2-NF-κB and RIG-I/MDA5-mitochondrial antiviral signaling protein (MAVS)-IRF3 pathways, and determine whether MDP plays an equivalent inhibitory role in both inflammatory pathways.

This investigation examined the anti-inflammatory effects of MDP and attempted to identify which of its active components most effectively contributes to those effects. We also investigated dose-dependent immunomodulatory activities of MDP and its derivatives on cytokine production. The in vivo effects of MDP and PGG were verified in the dextran sodium sulfate (DSS)-induced colitis mouse model.

## Results

### MDP water extract (WE) inhibits NF-κB and IRF reporters and downstream cytokine production

**Figure 1:**
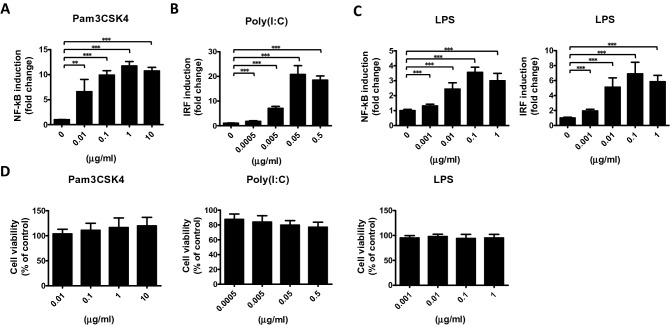
Dose-dependent responses of NF-κB or IRF reporter for various TLR agonists. THP1-Dual cells were stimulated with 0.01–10 μg/ml of the TLR2 ligand Pam3CSK4 (**A**), with 0.0005–0.5 μg/ml of the RIG-I/MDA5 ligand, transfected poly(I:C) (**B**), or 0.001–1 μg/ml of the TLR4 ligand LPS (**C**), as indicated at the top of each figure. After stimulation for 24 h, NF-κB or IRF activities were quantified. (**D**) An MTS assay determined the viability of THP1-Dual cells. Data are presented as the mean ± S.D (*n* = 5) and analyzed using one-way ANOVA. **p < 0.01, ***p < 0.001.

**Figure 2:**
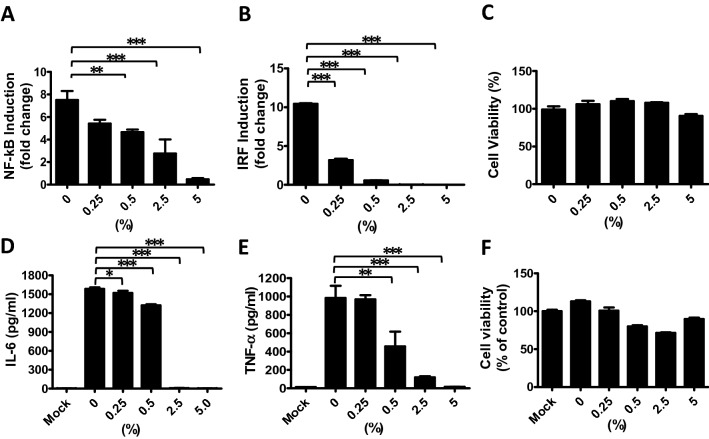
WE inhibited NF-κB and IRF promoter activation and downstream cytokine production. THP1-Dual cells or BMDMs were pretreated with WE (0, 0.25, 0.5, 2.5, or 5%) for 6 h then stimulated for 24 h with Pam3CSK4 for NF-κB activation, transfected poly(I:C) for IRF activation, or LPS for cytokine production. NF-κB (**A**) and IRF (**B**) activities were quantified. Changes from baseline were calculated as -fold changes by normalizing against the activity of unstimulated control cells. (**C**) Viability of THP1-Dual cells treated with different concentrations of WE was analyzed using the MTS assay. IL-6 (**D**) and TNF-α (**E**) production and viability (**F**) of BMDMs pretreated with WE were measured after 24 h of LPS stimulation. Mock cells were BMDMs without LPS stimulation. Data are presented as the mean ± S.D (*n* = 5) and analyzed using one-way ANOVA. **p < 0.01, ***p < 0.001.

We have previously prepared WE (described in the Materials and Methods section) and used HPLC-based mass spectrometry to confirm its chemoprofiles, which revealed well-known and important anti-inflammatory compounds, including paeonol, paeoniflorin, gallic acid, and PGG^13^. In order to demonstrate the anti-inflammatory effects of MDP, we established a cell-based screening system, in which NF-κB and IRF-inducible reporters were used as indicators for inflammatory responses. The monocytic cell line THP-1, which stably expresses two inducible reporter constructs, was used as the reporter cell line as previously described^23^, enabling verification of the inflammatory response by different TLR agonists (Fig. 1). NF-κB was stimulated specifically by a TLR2 ligand, Pam3CSK4, while IRF was activated by a RIG-I/MDA5 ligand, transfected poly(I:C). After undergoing stimulation for 24 h, NF-κB and IRF activities were dose-dependently enhanced (Fig. 1A,B). Similarly, both NF-κB and IRF reporters were activated in a dose-dependent manner by LPS, a TLR4 ligand (Fig. 1C). No cellular death occurred under these conditions (Fig. 1D). Next, the cells were pretreated with different amounts of WE for 6 h, before being stimulated with ligands. The half-maximal inhibitory concentration (IC_50_) of WE was around 2.5% (6.25 mg MDP residue per ml) for NF-κB and below 0.5% (1.25 mg MDP residue per ml) for IRF reporter inhibition (Fig. 2A,B), suggesting that WE strongly inhibits NF-κB and IRF promoter activity. No significant levels of cell death were detected under treatment with the indicated MDP concentrations (Fig. 2C). Given that MDP can strongly prohibit inflammatory promoter activity, inflammatory cytokines downstream of NF-κB were quantified. After LPS stimulation, levels of IL-6 and TNF-α secreted from bone marrow-derived macrophages (BMDMs) were substantially reduced under MDP treatment, in a dose-dependent manner (Fig. 2D,E), with no significant differences in levels of cell death (Fig. 2F). NF-κB and IRF signaling, as well as cytokine production, were consistently inhibited by treatment with WE.

### Of all compounds identified from MDP, PGG proved to be most effective at inhibiting NF-κB and IRF reporters and reducing inflammatory cytokine production

**Figure 3:**
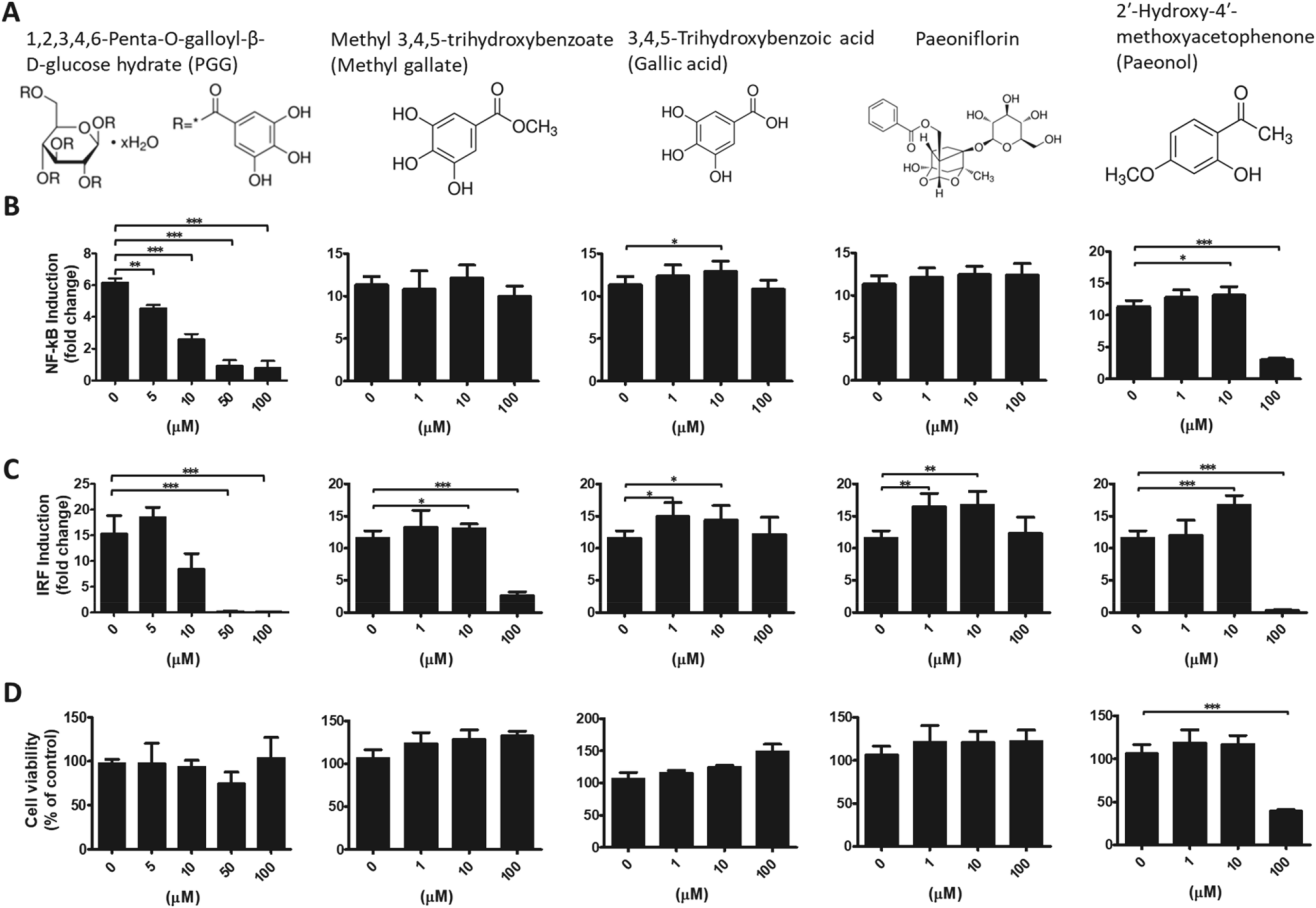
Inhibitory effects of MDP compounds on NF-kB and IRF reporters. (**A**) Chemical structures of identified MDP compounds. (**B**, **C**), THP1-Dual cells were pretreated with identified compounds of MDP (PGG, methyl gallate, gallic acid, paeoniflorin, paeonol) at different concentrations (1–100 μM) for 6 h then stimulated for 24 h with the TLR2 ligand, Pam3CSK4, for NF-κB activation (**B**), or the RIG-I/MDA5 ligand, transfected poly(I:C), for IRF activation (**C**). (**D**) The MTT assay determined the viability of THP1-Dual cells treated with different drug concentrations and stimulated by Pam3CSK4. Data are presented as the mean ± S.D (*n* = 5) and analyzed using one-way ANOVA. *p < 0.05, **p < 0.01, ***p < 0.001.

To identify which compound contributes the most to the anti-inflammatory effects of MDP, we screened MDP compounds that are known to possess anti-inflammatory activity, including methyl gallate, gallic acid, paeoniflorin, paeonol, and PGG. NF-κB and IRF reporter activities and chemical structures of each compound are shown in Fig. 3. PGG displayed the highest level of inhibitory behavior, with an IC_50_ of 5–10 µM for both NF-κB and IRF promoter activity (Fig. 3B,C). In contrast, the IC_50_ value of methyl gallate was 10–100 µM and was only observed with IRF promoter activity, with no significant levels of cell death under these concentrations (Fig. 3D). All other compounds, including gallic acid, paeonol and paeoniflorin, failed to display any inhibitory activity with either reporter at a very high concentration (100 μM) without cell death.

**Figure 4:**
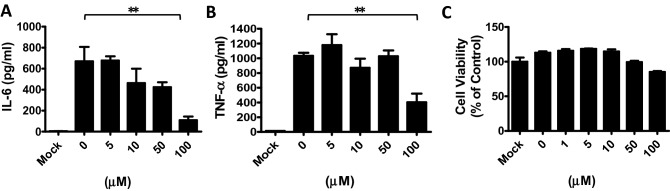
PGG inhibits NF-κB and IRF downstream cytokine production in BMDMs. BMDMs were pretreated with PGG (0–100 μM) for 6 h then stimulated with LPS for cytokine production. The mock cells were BMDMs without LPS stimulation. IL-6 (**A**) and TNF-α (**B**) production and viability (**C**) of BMDMs were measured after 24 h of LPS stimulation. Data are presented as the mean ± S.D (*n* = 5) and analyzed using one-way ANOVA. **p < 0.01, ***p < 0.001.

Among the compounds we tested, PGG proved to be the most effective at reducing both NF-κB and IRF reporter activation. Consequently, inflammatory cytokines were measured downstream of NF-κB. Secretion of IL-6 and TNF-α from BMDMs was significantly reduced under high concentrations of PGG treatment, i.e., at ~ 100 µM (Fig. 4A,B), with limited levels of cell death (Fig. 4C), suggesting that PGG alleviates NF-κB and IRF reporter activation and inflammatory cytokine production.

### Inhibitory effects of cellular metabolites of PGG and WE on NF-κB and IRF reporters

**Figure 5:**
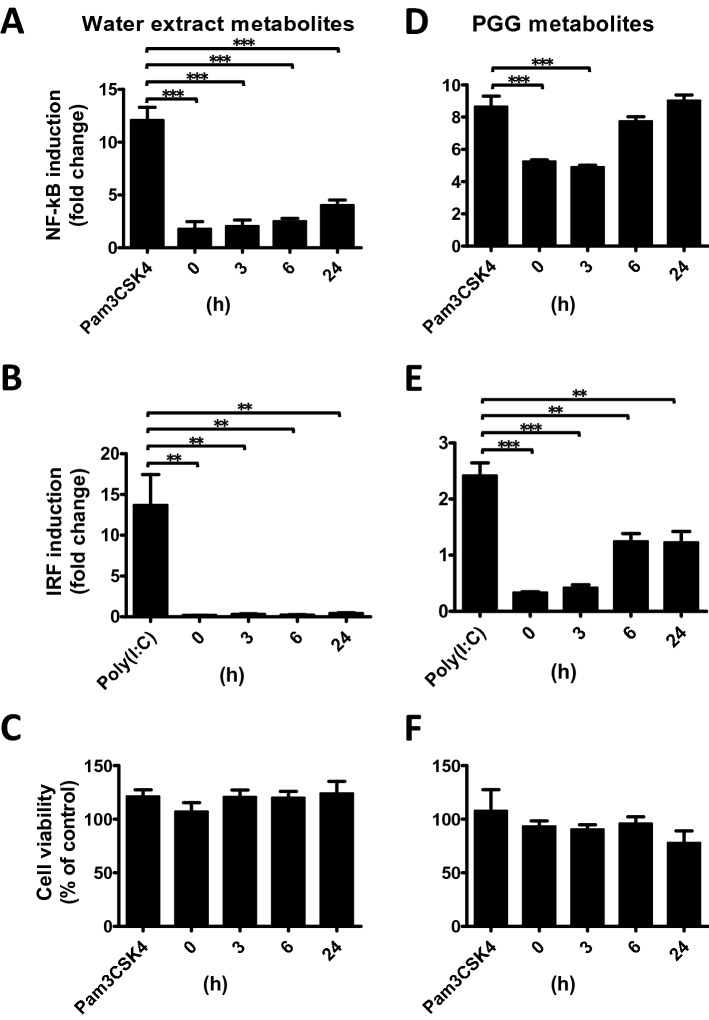
Inhibitory effects of metabolites from PGG and MDP on NF-kB and IRF reporters. THP1-Dual cells were treated with conditioned medium obtained from supernatants of cells treated with 50 uM PGG or 0.5% WE for different time periods (0, 3, 6, or 24 h), then stimulated with Pam3CSK4 or poly(I:C) for 24 h and NF-κB (**A**, **D**) and IRF (**B**, **E**) reporter activities were measured. (**C**, **F**) The MTS assay determined the viability of THP1-Dual cells subjected to metabolite and Pam3CSK4 stimulation. Control cells labeled with Pam3CSK4 or poly(I:C) underwent stimulation only. Data are presented as the mean ± S.D (*n* = 3) and analyzed using one-way ANOVA. *p < 0.05, **p < 0.01, ***p < 0.001.

It is possible that the efficacy of drug treatment upon the cells arose from the metabolites of the drug and not the drug per se. To verify this possibility, we collected conditioned medium from cells treated for different time periods with 50 uM PGG or 0.5% WE and tested the inhibitory effects of the cellular metabolites in the medium on IRF promoter activity. Metabolites of WE slightly reduced the inhibitory activity of NF-κB (Fig. 5A) but not of IRF reporters (Fig. 5B) without significant cell death (Fig. 5C), suggesting that the active compounds may not be metabolites of MDP. In contrast, PGG strongly reduced the inhibitory activity of both NF-κB and IRF promoters (Fig. 5D–F), implying that PGG or its metabolites are easily degraded into inactive components or are very unstable in cell culture conditions.

### WE but not PGG strongly inhibits NF-κB and IRF signaling pathways

**Figure 6:**
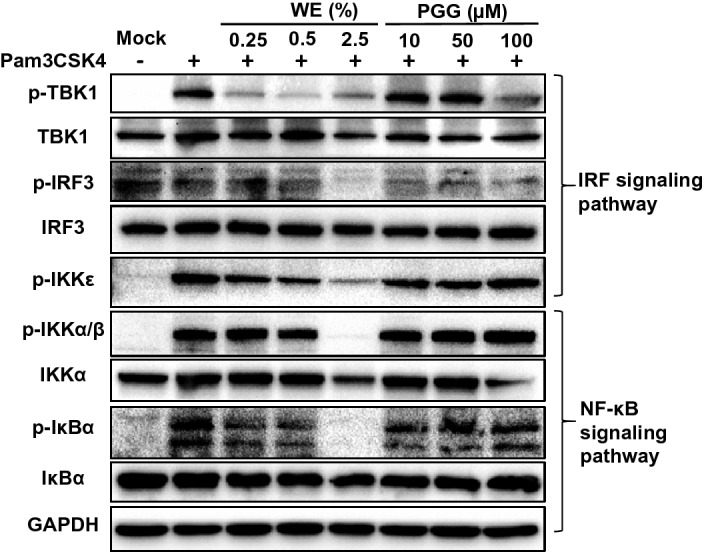
WE but not PGG strongly inhibits NF-κB and IRF signaling pathways. BMDMs were pretreated with water extract (0.25, 0.5, or 2.5%) or PGG (10, 50, or 100 μM) for 2 h. Activation of cells with Pam3CSK4 stimulation for 1 h are shown by immunoblots using indicated antibodies. Full-length blots and markers are included in the Supplementary Fig. S2.

In order to demonstrate the anti-inflammatory effects of MDP and compare the efficacy of MDP and PGG, we used immunoblots to verify the signaling pathways induced by Pam3CSK4, a TLR2 ligand (Fig. 6). While primary macrophages were activated by Pam3CSK4, the NF-κB and IRF signaling pathways were both inhibited in cells treated with WE (Fig. 6). The 2.5% solution of WE, which contains 0.025 μM PGG, completely abolished IRF3, TBK, IKKα/β and IκBα phosphorylation and activation. Given that only a high concentration of synthetic PGG (100 μM) can partially inhibit IRF but not NF-κB signaling pathways (Fig. 6), WE clearly has superior anti-inflammatory effects in both pathways. Consistent with our reporter and cytokine production assays, WE effectively alleviated TLR-2 mediated IRF and NF-κB signaling pathways.

### MDP and its water extract, but not PGG, reduce inflammation in the DSS-induced mouse colitis model

**Figure 7:**
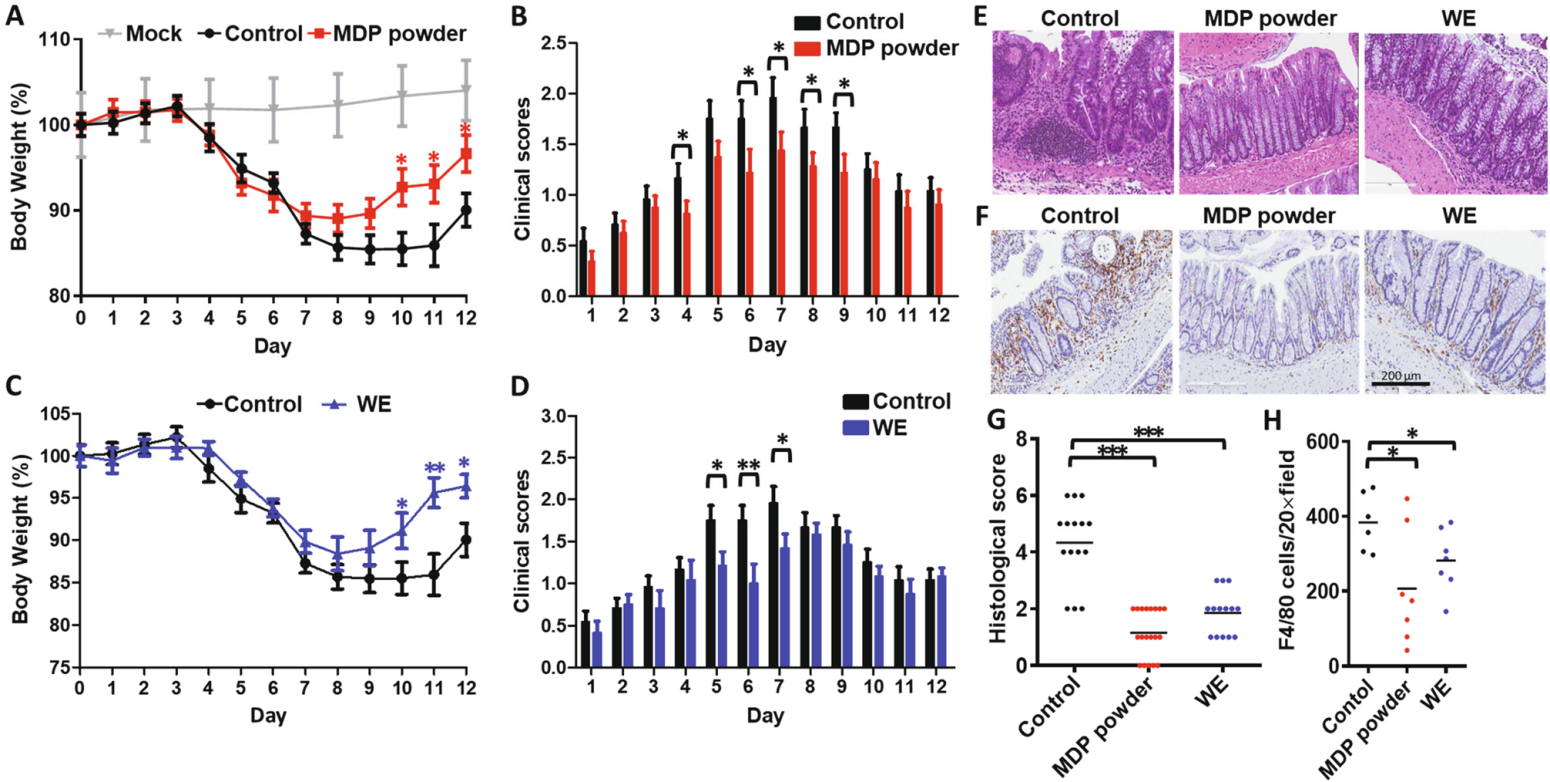
MDP powder and WE alleviated DSS-induced colitis in mice. (**A**–**D**) Mice were treated orally by gavage with MDP powder (**A**, **B**) or WE (**C**, **D**) and colitis was induced with 3% DSS for 5 days (*n* = 10–11 per group). Mock, mice without DSS treatment (*n* = 7). (**A**, **C**) Percentage of body weight. (**B**, **D**) Clinical scores. (**E**, **F**) Colon samples from mice were collected on day 12 (*n* = 6–10 per group). Images (**E**) and semiquantitative scoring (**G**) of hematoxylin and eosin-stained colon sections. Images (**H**) and quantification (**F**) of F4/80 positive cells in colon sections are shown by immunohistochemistry. Data are presented as the mean ± S.E.M. and analyzed using the Student's *t*‐test compared with the control group. *p < 0.05, **p < 0.01, ***p < 0.001.

Given that MDP has strong anti-inflammatory effects, we examined whether WE and PGG treat IBD in mice. We applied two different MDP forms in our colitis model (Fig. 7); MDP powder for clinical use purchased directly from the manufacturing drug company, and WE. Mice were provided with MDP or WE daily by oral gavage and colitis was induced by adding 3% DSS to the drinking water for 5 days, before changing to normal water for 7 days. Body weight percentages and clinical scores were examined daily. During days 10 to 12, both MDP and WE mitigated the effects of DSS-induced colitis, as evidenced by smaller amounts of body weight loss (Fig. 7A,C) and lower clinical inflammation scores at earlier time points (Fig. 7B,D), compared with control mice. Hematoxylin and eosin staining of colonic sections demonstrated less colonic inflammation in the mice treated with MDP or WE versus controls, with lower quantities of tissue-infiltrated immune cells compared with mice in the control group (Fig. 7E). Pathological scores of tissue integrity were also higher after MDP and WE treatments compared with scores in the control group (Fig. 7G). Consistent with our previous finding that WE inhibited the activation of BMDMs, tissue-infiltrated macrophages stained by F4/80 were also reduced in mice with MDP and water extract versus controls (Fig. 7F,H), which suggested reductions in the levels of activated macrophages after treatment. Body weight and clinical inflammation scores were not significantly changed from baseline in mice orally gavaged with PGG (Supplementary Fig. S1A,B), suggesting that PGG has weak inhibitory effects on macrophages in vivo. All findings suggested that MDP powder and its water extract are highly effective at resolving immune cell activation and infiltration in the gut, reducing colitis severity in a murine model.

## Discussion

Of the many TCM substances that are used for treating IBD, one of the most common is MDP. This substance can serve as a heat-clearing and blood-cooling agent, promoting blood circulation and dissipating blood stasis, so TCM clinicians often use MDP to treat inflammatory diseases^24^. MDP can be prescribed as a single herb or in combination with other TCM agents. The well-known Chinese herbal formulas Guizhi Fuling Formula^25^, Liu Wei Di Huang Wan (also administered as the Liu-Wei-Di-Huang pill)^26^, and Jia-Wei-Xiao-Yao-San (also known as Dan-Zi-Xiao-Yao-San) all contain MDP. Guizhi Fuling Formula is a common treatment for several female illnesses, such as masses in the abdomen, amenorrhea caused by blood stasis, menses with belly ache, and persistent lochia after child delivery where MDP is used to promote blood circulation and dissipates blood stasis. MDP in Guizhi Fuling Formula is also used for anti-inflammatory conditions, including chronic pelvic inflammatory disease, endometritis, oophoritis, salpingitis, uterine fibroids, women's menopausal syndrome, menstrual abnormalities, chronic cervicitis, and leucorrhea. Liu Wei Di Huang Wan is a common treatment for yin deficiency syndrome, such as dryness of mouth, low back pain and dizzy where MDP is used to clear excessive heat. In contrast, Jia-Wei-Xiao-Yao-San, also known as Dan-Zi-Xiao-Yao-San, also includes MDP and is used to promote relief of *Qi* stagnation, heat-clearing, and blood-cooling. The adverse effect of excessive heat-clearing associated with MDP is neutralized by other components in the formulas.

The active compounds of these TCMs have yet to be identified and their molecular mechanisms remain unclear. In this investigation, the water extract of MDP shows very strong inhibitory activity on macrophage activation by targeting TLRs, RIG-I/MDA5, and downstream signaling. Our screening of known MDP compounds with anti-inflammatory activity identified the most effective active compound to be PGG, a polyphenolic bioactive constituent of many medicinal plants. PGG has been verified in many disease models, including kidney injury^20^, cancer^27^ and viral infections^28^. It is known that PGG prevents the phosphorylation of cytosolic IκB proteins and thus alleviates the TLR-NF-κB signaling pathway^29,30^. By dissecting the TLR-NF-κB and RIG-I/MDA5-IRF3 pathways, we found that MDP plays important roles not only in preventing NF-κB activation, but also in regulating the IRF pathway. The inhibitory effects of MDP and PGG on these two pathways are not equivalent. Compared to the limited activities of PGG in both pathways, MDP more efficiently inhibits IRF reporter activity compared with NF-κB activity. Since IRF is important for type I IFN induction, MDP may be useful in type I IFN-dominant diseases, such as viral infections.

It has been reported that many compounds identified from MDP have anti-inflammatory activities. However, in our screening system, many of them displayed very little or no effect on both NF-κB and IRF reporters, or exhibited inhibitory qualities only at very high concentrations, such as synthetic PGG, which had an IC_50_ of ~ 10 μM (Fig. 3). In our previous publication, the PGG concentration in WE was measured as 975.90 ± 14.10 ppb (1.04 ± 0.02 μM)^13^. The amount of PGG in the working concentration of WE was ~ 25 nM and much lower than the IC_50_ of synthetic PGG compound, suggesting that PGG may not be the major contributor in MDP anti-inflammatory activity. Because MDP powder and WE were obtained from different preparations, it is difficult to compare the efficacy of MDP powder with that of WE in the DSS-induced colitis model (Fig. 7). However, both TCM preparations derived from MDP demonstrated therapeutic effects in colitis, suggesting that both have therapeutic activities. In reference to our measurement of concentrations of PGG in WE^13^, the amount of PGG in WE that we gave to mice was 1.18 mg/kg (Fig. 7), which is much lower than the amount of synthetic PGG compound (20 mg/kg) in the colitis model (Fig. S1). Evidence from both cellular (Figs. 3 and 6) and animal experiments (Fig. 7) clearly reveal that PGG is not the key compound that independently reduces inflammatory responses. In contrast to PGG, the strong inhibitory activity of WE, which contains relatively low concentrations of individual compounds, has therapeutic effects. This effect may either be the result of a synergistic effect of multiple compounds, or is due to an as-yet unidentified compound. To fully understand the immunomodulating mechanisms underlying MDP, our future studies will seek to identify as-yet unknown components in MDP that can inhibit these two pathways and determine the maximum synergistic effects by reconstituting and combining individual compounds.

In this study, WE significantly inhibited NF-κB and IRF reporters and alleviated downstream IL-6 and TNF-α production in a dose-dependent manner, without inducing cell death. Among the five known active components identified from MDP, PGG was the most efficient in this study in inhibiting NF-κB and IRF reporters (with half-maximal inhibitory concentrations [IC_50_] of 5–10 µM in each condition) and in downregulating production of the proinflammatory cytokines. Methyl gallate inhibited only the IRF promoter at a very high concentration (100 µM). At 100 μM, all other compounds, including gallic acid, paeonol, and paeoniflorin failed to inhibit either the NF-κB or the IRF reporter. MDP powder and WE, but not PGG, reduced colon inflammation in the DSS-induced colitis model. All of our study findings suggest that MDP powder and its water extract are highly effective at resolving immune cell activation and infiltration in the gut, and thereby reducing colitis severity. Thus, MDP and its water extract offer promise as a novel therapeutic strategy for treating inflammatory diseases and IBD.

## Materials and methods

### Antibodies and reagents

Antibodies used in this study were F4/80 (Cat. MCA497G, Lot 1806, Bio-Rad, Hercules, CA, USA; 1:200) and glyceraldehyde 3-phosphate dehydrogenase (Cat. 100118, Lot 42928, GeneTex; 1:5,000). All other antibodies came from Cell Signaling (1:2000), including p-TBK1 (Cat. 5483, Lot 11), TBK1 (Cat. 3504, Lot 4), p-IRF3 (Cat. 29047, Lot 4), IRF3 (Cat. 4302, Lot 4), p-IKKε (Cat. 8766, Lot 3), p-IKKα/β (Cat. 2697, Lot 16), IKKα (Cat. 27609), p-IκBα (Cat. 2859, Lot 18), IκBα (Cat. 4814, Lot 17). LPS, poly(I:C) and Pam3CSK4 were obtained from Invivogen. PGG, methyl gallate, gallic acid, paeoniflorin, and paeonol were purchased from Sigma (Sigma-Aldrich).

### MDP powder and preparation of WE

MDP powder was purchased as a commercialized powder form for clinical use from Sun Ten Pharmaceutical Company, Ltd. (Taipei, Taiwan). WE was prepared by the same company following our requested procedures as follows: 100 g *P. suffruticosa* was boiled with 1.5 L water at 100 °C for 30 min, then concentrated to 100 ml under reduced pressure. A clear supernatant (WE) was further processed by centrifugation at 14,000×*g* for 20 min, which contained the weight of a total of 250 mg residue per ml after dehydration in vacuo. WE components were examined by liquid chromatography tandem-mass spectrometry (LC–MS/MS), using previously described methods^13^.

### Cells and culture conditions

THP1-Dual cells were purchased from InvivoGen and cultured according to the manufacturer’s manual. BMDMs were isolated from mice and differentiated for 5–6 days, following standard procedures^31^. Adherent BMDMs were cultured overnight at a density of 1 × 10^6^ cells per ml before being treated with 5, 2.5, 0.5, or 0.25% WE (equivalent to 12.5, 6.25, 1.25, or 0.625 mg MDP residue per ml, respectively) or with the indicated chemicals for 6 h. The cells were then stimulated with TLR ligands at the indicated concentrations^32^.

### Reporter assays

THP1-Dual cells were pretreated with WE or indicated chemicals for 6 h, then stimulated with TLR2 ligand (0.1 μg/ml Pam3CSK4) or RIG-I/MDA5 ligand (0.05 μg/ml poly(I:C)/LyoVec), following the manual’s procedures. After undergoing stimulation for 24 h, NF-κB and IRF induction was quantified by measuring the levels of secreted alkaline phosphatase (SEAP) and luciferase activity in the culture supernatant using QUANTI-Blue (InvivoGen) and QUANTI-Luc (InvivoGen), respectively. Changes from baseline were calculated as -fold changes, normalized against unstimulated control cells.

### Viability assays

The viability of THP1-Dual cells was analyzed using the MTS assay. THP1-Dual cells were pretreated with water extract or chemicals before RIG-I/MDA5 ligand stimulation. After stimulation for the indicated times, the cells were washed twice with fresh medium. Cell viability was analyzed using an MTS assay kit (Abcam). After 2 h of incubation, cell viability was determined by the amount of 490 nm absorbance. The percentage of cell death was calculated by normalization with cells that were either untreated or treated with the ligand only.

### DSS-induced colitis model

The same aged-, sex- and weight-matched C57BL/6N mice were kept in a pathogen-free environment and were aged 8–12 weeks at the time of the experiments. DSS-induced colitis was induced by the administration of 3% DSS (36,000–50,000 molecular weight; MP Biomedicals, Santa Ana, CA, USA) in the drinking water. DSS drinking water was made available to the mice for 5 days, followed by 7 days of normal water, as previously reported^33^. In the drug treatment experiment, mice were randomly assigned to one of two groups; the control (vehicle) group and the treatment group. In the treatment group, mice were fed daily with MDP (20 mg/kg), MDP water extract (312.5 mg MDP residue/kg), or PGG (20 mg/kg), starting at the same time as DSS treatment. MDP was purchased from Sun Ten Pharmaceutical Company and its clinical dose suggested by the company was 20 mg/kg. Body weights and stool samples were monitored daily starting from day 0 of DSS treatment, as previously described^34^. The baseline clinical score was determined at the moment DSS treatment was started (day 0). Scoring for stool consistency and occult blood was done as described previously^34^. Briefly, stool scores were determined as follows: 0 = well-formed pellets, 1 = semi-formed stools that did not adhere to the anus, 2 = semi-formed stools that adhered to the anus, 3 = liquid stools that adhered to the anus. Bleeding scores were determined as follows: 0 = no blood by using Hemoccult SENSA (Beckman Coulter), 1 = positive Hemoccult SENSA, 2 = visible blood traces in stool, 3 = rectal bleeding. The stool and bleeding scores were averaged to calculate the clinical score. On day 12, the colon was removed, measured, photographed, and fixed in 10% formalin. Intestinal inflammation was assessed histologically using previously described methods^33^. The histological scores were determined as follows: 0 = presence of occasional inflammatory cells in the lamina propria, 1 = increased numbers of inflammatory cells in the lamina propria, 2 = confluence of inflammatory cells extending into the submucosa, 3 = transmural extension of the infiltrated cells. Tissue damage was scored as follows: 0 = no mucosal damage, 1 = lymphoepithelial lesions, 2 = surface mucosal erosion or focal ulceration, 3 = extensive mucosal damage and extensive deeper structural damage. The combined histological score ranged from 0 (no changes) to 6 (extensive infiltrated cells and tissue damage). All animal care and experimental methods were approved by the Institutional Animal Care and Use Committee of China Medical University, Taichung, Taiwan and were performed in line with the Guideline for the Care and Use of Laboratory Animals and approved by Taiwan’s government organization, Council of Agriculture, Executive Yuan.

### Enzyme-linked immunosorbent assay (ELISA)

Cytokine kits for detecting mouse IL-6 and TNF-α were obtained from eBioscience. Cytokine measurements were performed according to their respective manuals provided by eBioscience.

### Statistics

Results are presented as the mean ± S.E.M. in mouse model experiments and as the mean ± S.D. in experiments using primary macrophages or THP-1 cells. Data were analyzed using the Student’s *t*‐test (two-tailed) or one-way ANOVA with the Tukey's multiple comparison test. A *p*-value of < 0.05 was considered significant.

## Supplementary information

Supplementary Information.
