# Supplementary material for: A systematic identification of anti-inflammatory active components derived from Mu Dan Pi and their applications in inflammatory bowel disease
Source: Sci Rep. 2020 Oct 14;10:17238. doi: 10.1038/s41598-020-74201-x (PMC7560859; doi:10.1038/s41598-020-74201-x)
Supplement: Supplementary file 1 — Supplementary Information. [file 41598_2020_74201_MOESM1_ESM.pdf]

Supplementary information

A systematic identification of anti-inflammatory active components derived from Mu Dan Pi and their applications in inflammatory bowel disease

Tzu-Fan Chen, Jeh-Ting Hsu, Kun-Chang Wu, Che-Fang Hsiao, Jou-An Lin, Yun-Hsin Cheng, Yu-Huei Liu, Der-Yen Lee, Hen-Hong Chang, Der-Yang Cho, and Jye-Lin Hsu

Supplementary figure legends

Supplementary Fig. S1. PGG had no effect on DSS-induced colitis in mice. Mice were orally gavaged with PGG (20 mg/kg) daily and colitis was induced with 3% DSS for 5 days (*n* = 10–11 per group). Percentages of body weight (A) and clinical scores (B) of mice were measured as described in the methods.

Supplementary Fig. S2. Full-length immunoblots and markers in the Fig. 6.

Fig. S1

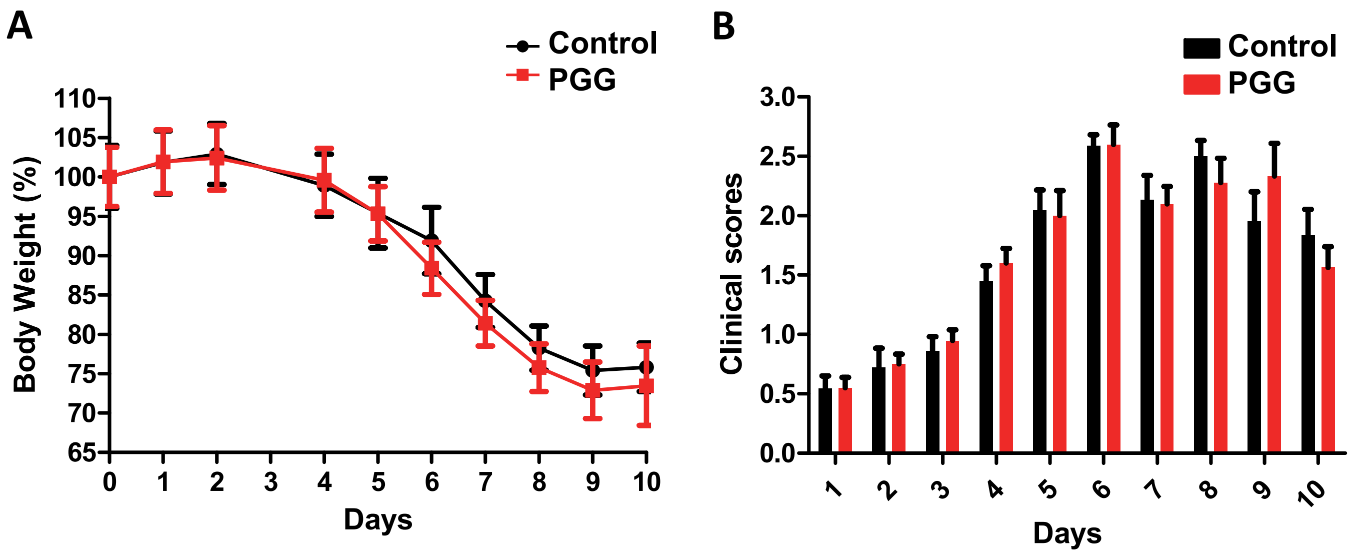
A
Control
PGG
110
105
100
95
90
85
80
75
70
65
Body Weight (%)
0
1
2
3
4
5
6
7
8
9
10
Days
B
Control
PGG
3.0
2.5
2.0
1.5
1.0
0.5
0.0
Clinical scores
1
2
3
4
5
6
7
8
9
10
Days

Fig. S2

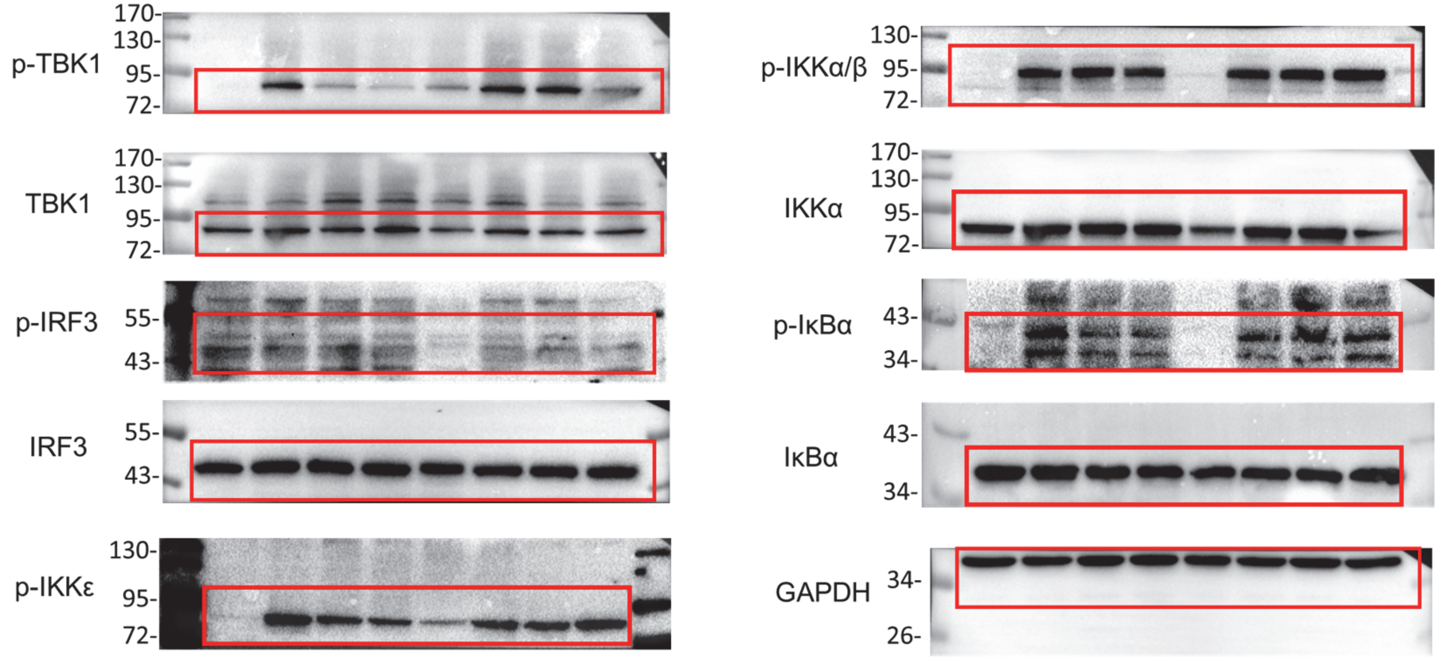

p-TBK1
170-
130-
95-
72-
TBK1
170-
130-
95-
72-
p-IRF3
55-
43-
IRF3
55-
43-
p-IKKε
130-
95-
72-
p-IKKα/β
130-
95-
72-
IKKα
170-
130-
95-
72-
p-IκBα
43-
34-
IκBα
43-
34-
GAPDH
34-
26-
